# A Screening Strategy for Metal Antitumor Agents as Exemplified by Gold(III) Complexes

**DOI:** 10.1155/MBD.1999.291

**Published:** 1999

**Authors:** Simon P. Fricker

**Affiliations:** AnorMED Inc. #100 20353-64th Avenue BC Langley V2Y 1N5 Canada

## Abstract

The use of a screening strategy based on human tumor cell lines, which are also tumorigenic in immune-deprived mice, is described. Activity against the human tumor cells *in vitro* and *in vivo*, is complemented with studies on the mechanism of action. This aporoach is demonstrated using 2-[(dimethylamino)-methyl]phenyl (damp) gold(III) compounds of the type [Au X2(damp)], where X = chloride, thiocyanate, acetate, or the bidentate ligands oxalate and malonate. All compounds were shown to be selectively active against a human bladder tumor cell line (HT1376) *in vitro*, and active in an *in vitro* panel of ovarian tumor cell lines. The acetato complex was shown to be active in experimental animal models of both the HT1376 bladder tumor, and the CH1 ovarian tumor. Although the [AuX2(damp)] compounds share structural features in common with cisplatin, and exhibit interesting *in vivo* activity against human tumor cells, the data indicate that they have a very different biochemical mechanism from platinum-based drugs and represent a potentially new class of metal-based antitumor agents.


### A SCREENING STRATEGY FOR METAL ANTITUMOR AGENTS AS EXEMPLIFIED BY GOLD(III) COMPLEXES

Simon P. Fricker

AnorMED Inc. #100, 20353-$64^{th}$ Avenue, Langley, BC, V2Y 1N5, Canada

The use of a screening strategy based on human tumor cell lines, which are also tumorigenic in immune-deprived mice, is described. Activity against the human tumor cells *in vitro* and *in vivo*, is complemented with studies on the mechanism of action. This approach is demonstrated using 2-[(dimethylamino)-methyl]phenyl (damp) gold(III) compounds of the type [Au X2(damp)], where X = chloride, thiocyanate, acetate, or the bidentate ligands oxalate and malonate. All compounds were shown to be selectively active against a human bladder tumor cell line (HT1376) *in vitro*, and active in an *in vitro* panel of ovarian tumor cell lines. The acetato complex was shown to be active in experimental animal models of both the HT1376 bladder tumor, and the CH1 ovarian tumor. Although the [AuX2(damp)] compounds share structural features in common with cisplatin, and exhibit interesting *in vivo* activity against human tumor cells, the data indicate that they have a very different biochemical mechanism from platinum-based drugs and represent a potentially new class of metal-based antitumor agents.

**Introduction: An Historical Perspective**

Strategies for identifying new anticancer agents have developed and evolved over the years from simple animal models to the incorporation of the latest techniques in molecular biology. Historically however these strategies have been predominantly based on *in vivo* rodent tumor models.[1] The earliest rodent models that were used, prior to 1955, were allogeneic transplantable tumors such as Sarcoma 180, Erlich ascites, Yoshida ascites sarcoma, and the Walker 256 carcinoma. These tumors are rapidly growing, undifferentiated cell types. Being allogeneic the host animal will frequently mount an immune response to the tumor, also the solid tumors such as Sarcoma 180 are frequently sensitive to host weight loss. Both of these factors make it difficult to differentiate drug effect from host effects. In addition some of these tumors such as the Erlich ascites are very sensitive to cytotoxic agents thus leading to large numbers of false positives in the screen.

As a result of these limitations the allogeneic tumor models were superseded by syngeneic transplantable murine tumors such as the L1210 and P388 leukemia's, and the Lewis Lung carcinoma, and B16 melanoma. These tumors retain their histologic type on transplantation, grow rapidly and reproducibly, and give reproducible results with control drugs, therefore satisfying many of the requirements of a screening system. They have been used extensively by many laboratories for the pre-clinical selection of new antitumor agents.

One of the most well documented screening programs has been that of the American National Cancer Institute.[1] Between 1955 and 1975 their primary screening system consisted of the L1210 and P388 leukemia's, supplemented with other rodent tumors including the B16 melanoma and the Lewis Lung carcinoma.[2] In 1975 they introduced a two-tier system to broaden the number of tumor types against which new agents could be screened. This involved a pre-primary screen using the P388 leukemia followed by a primary tumor panel including murine tumors and human xenografts. This was used until 1990 with a few variations on the theme. In spite of the inclusion of other tumor models in the screening panel in practice the majority of compounds were only screened against the murine leukemias. The outcome of this screening strategy was the discovery of a number of agents that were successfully introduced into the clinic for the treatment of leukemias. There was, however, only limited success in identifying agents for the treatment of the most commonly occurring, major solid tumors of lung, breast and colon. The NCI experience has been reflected in that of other cancer institutes and laboratories worldwide.[3]

**Table 1** Historical summary of cancer drug screening

| YEAR | PRIMARY SCREEN | SECONDARY SCREEN |
|---|---|---|
| Pre 1955 | Allogeneic transplantable murine tumors:<br>Sarcoma 180<br>Erlich Ascites<br>Yoshida ascites sarcoma<br>Walker 256 carcinoma | |
| 1955 | Walker 256 carcinoma<br>Syngeneic transplantable murine tumors:<br>L1210 leukemia | |
| 1968 | L1210 murine leukemia<br>P388 murine leukemia | |
| 1972 | L1210 and P388 leukemia's<br>B16 melanoma<br>Lewis lung carcinoma | |
| 1975 | P388 leukemia | Murine tumors:<br>L1210 leukemia<br>B16 melanoma<br>Lewis lung<br>Xenografts:<br>MX-1, CX-1, LX-1 |
| 1982 | P388 leukemia | Murine tumors:<br>L1210, B16, M5076, LL<br>Xenografts: MX-1 |
| 1990 | Re-evaluation | |

**Alternative Strategies**

The limited success in identifying agents for the treatment of solid tumors led to a major re-evaluation of screening strategies for new cancer drugs. One possible explanation proposed for this failure was the predominant use of murine leukemias as a primary screen. There are two major limitations to the murine leukemias as tumor models. One is that the cancer cells and test drug are in close proximity. The leukemia cells are grown as ascitic suspensions in the peritoneal cavity of the host animal and test compounds are usually administered directly into the peritoneal cavity, whereas in contrast solid tumors are distant from the site of drug administration. In consequence in these simple ascitic models there is no need to take into account the barriers a drug has to face in reaching its target, both in terms of distribution and metabolism. Secondly, being a mouse leukemia, the cell type is not typical of a human solid tumor, which will have different characteristics and susceptibilities to drugs. This latter problem has been the focus of attention in a number of laboratories, including the NCI. They have adopted a system based on panels of human tumor-derived cell lines. This is a disease oriented approach utilising a panel of 60 cell lines derived from commonly occurring tumors including melanomas, leukemias, breast, prostrate, lung, colon, ovary, kidney, and central nervous system.[4] These cell lines are therefore representative of the different histologic types of many solid tumors. The hypothesis is that compounds with selective activity against cell lines of a particular tissue type would be predictive for activity against the corresponding tumor. The cell lines are challenged *in vitro* with the test agent and cell growth monitored using a colorimetric assay which measures total cell protein using an aminoxanthene dye, sulforhodamine B. The activity of test compounds is compared to that of known agents with defined mechanisms of action using a computer programme called COMPARE. The concept is still being evaluated as compounds progress through to clinical trials however it is becoming apparent that this approach is proving to be particularly useful in providing valuable insight into drug mechanism, and molecular targets within cancer cells.[5] A similar approach has been adopted by the EORTC in Europe,[3] the Institute of Cancer Research in the UK,[6] and our own laboratory.[7]

These are of course *in vitro* systems and therefore do not predict for the *in vivo* behaviour of a drug such as its pharmacokinetics, metabolism, or toxicity. An alternative to using *in vivo* murine cancers is to use xenografts of human cancer cells transplanted into immunedeprived mice. Many of the cell lines used in the *in vitro* panels described above can be grown as xenografts, therefore compounds selected in the *in vitro* screen can be further evaluated *in vivo* against the same tumor cells. Usually the tumor cells are implanted subcutaneously in the flank of the host animal where a solid tumor will form and grow. The test drug can then be administered by a number of routes. The intraperitoneal route is the most common route for drug screening, but compounds can also be administered orally or intravenously as appropriate. The effect of drug on tumor growth is monitored over a suitable time period, usually a minimum of 28 days.

An alternative to screening compounds in tumor models is to evaluate compounds against particular molecular and cellular targets.[8] This mechanistic approach to drug discovery can take a number of forms, depending upon the target. Cell signalling pathways responsible for transmitting information from the outside of a cell to the nucleus have become the research focus for a number of laboratories. Many of the proteins and enzymes implicated in these pathways are encoded by oncogenes, a typical example being the receptor-mediated tyrosine kinases. Many cancer drugs kill cancer cells by triggering programmed-cell death or apoptosis. The intracellular events responsible for controlling apoptosis are being studied with the view to gaining an understanding of how these events can be exploited in the development of new drugs. Other molecular targets include key enzymes in DNA metabolism and repair, and DNA itself.

One of the major problems facing oncologists is the spread of primary solid tumors to distant sites leading to the establishment of secondary tumors. This spread of tumors throughout the body, is a multi-step process, and is known as metastasis. Part of this process involves degradative enzymes including metalloproteases, cysteine proteases and the serine proteases.[9] These enzymes break down the basement membrane surrounding a solid tumor thus allowing tumor cells to migrate to distant sites within the body and establish secondary tumors. These secondary tumors are generally refractory to normal therapy. In order for both the primary secondary tumors to be established they have to develop a blood supply. This process of neovascularisation is known as angiogenesis.[10] Both metastasis and angiogenesis are key targets for new drug discovery.

The use of *in vitro* human tumor cell lines, *in vivo* xenografts of human tumors, and molecular mechanism and target directed screening are all proving to be valuable tools in the discovery process for novel anticancer drugs.

### Evaluation of Gold(III) Complexes

The discovery of the antitumor activity of cisplatin *cis*-$[PtCl_2(NH_3)_2]$ in 1969 prompted the continuing search for other metal-based drugs. Gold has been included in this search; and there has been intensive effort to identify a gold antitumor drug.[11,12] The main focus has been on complexes of gold(I), particularly gold phosphine complexes such as $[Au(dppe)_2]^+$ which showed promising activity in a number of tumor models. In our own laboratory we adopted an integrated approach to the search for new metal-based anticancer drugs, incorporating *in vitro* screening, *in vivo* human tumor xenograft models, and mechanistic studies. The value of this integrated approach is illustrated in a collaborative project with the Department of Chemistry, UMIST, UK and the Institute of Cancer Research, UK, investigating the potential antitumor activity of a class of gold(III) complexes ( see Parish, This volume pages_##).

Gold(III) is isoelectronic ($d^8$) with platinum(II) and likewise forms square planar complexes. It is therefore tempting to speculate that such complexes would have similar antitumor activity to cisplatin. Gold(III) complexes have not been as thoroughly investigated as gold(I) complexes, primarily because of their reactivity. In order to stabilise the gold(III) oxidation state in a reducing biological milieu we have synthesised complexes with a single mononegative bidentate ligand, damp, (2-[(dimethylamino)methyl]phenyl), and two monodentate anionic ligands e.g. Cl or acetate, or bidentate ligands such as oxalate and malonate (Figure 1).[13] The damp ligand forms part of a five-membered chelate ring in which the nitrogen of the amine group and the carbon of the aryl ring bond to the metal. The monodentate ligands are readily hydrolysed and are available for substitution.[14]

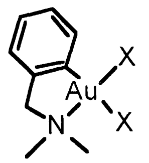

1: X=Cl

2: X=SCN

3: X=acetato

4: $X_2$=oxalato

5: $X_2$=malonato

**Figure 1** Structures of Au(III) 2-[(dimethylamino)methyl]phenyl complexes.

The compounds were first tested against two panels of human tumor cell lines. The primary cell panel consisted of cells from tumors representative of different tissue types and with different chemosensitivities to cisplatin (Table 2).[15] Differential cytotoxicity, as opposed to non-selective toxicity, was used as an indicator of potential antitumor actvity of test compounds.[7] The second panel was disease-oriented and consisted of cell lines established from human ovarian tumors. This panel included two pairs of cisplatin-sensitive and cisplatin-resistant lines, with defined mechanisms of resistance (CH1/CH1-R, A2780/A2780-R), and also two inherently resistant cell lines (HX62 and SK-OV-3). This panel has been used to identify agents with the ability to circumvent cisplatin resistance (Table 3).[6]

**Table 2** Selective cytotoxicity ($IC_{50}$ μM) of cisplatin to the primary cell line panel.

| SW620 COLON | SW1116 COLON | ZR-75-1 BREAST | HT29/219 COLON | HT1376 BLADDER | SK-OV-3 OVARIAN | RANGE (Lowest $IC_{50}$/Highest $IC_{50}$) |
|---|---|---|---|---|---|---|
| 167 | 163 | 27 | 17 | 23 | 23 | 9.8 |

**Table 3 Cytotoxicity ($IC_{50}$ μM) of cisplatin towards cisplatin sensitive and resistant ovarian cell lines. Mechanisms of resistance are shown, numbers in parentheses represent resistance factors of sensitive/resistant pairs ($IC_{50}$ of resistant cell line:$IC_{50}$ of sensitive cell line)**

| HX62 | SK-OV-3 | CH1 | CH1-R | A2780 | A2780-R | RANGE (Lowest $IC_{50}$/ Highest $IC_{50}$) |
|---|---|---|---|---|---|---|
| 18<br>Intracellular detoxification<br>Decreased drug accumulation | 5.2<br>Intracellular detoxification<br>DNA damage repair | 0.12 | 0.56 (4.7)<br>DNA damage repair | 1.2 | 10 (8.3)<br>Intracellular detoxification<br>DNA damage repair | 150 |

Most of the cell lines used can also be grown as solid tumor xenografts in immune-deprived, nude, mice and thus allowing the compounds to be further evaluated *in vivo* against the tumor types found to be sensitive *in vitro*. A typical tumor growth curve for the HT1376 bladder carcinoma is shown in Figure 2. The growth rate of this tumor is reduced by cisplatin at a dose of 4.5 mg/kg, the maximum tolerated dose for cisplatin in this tumor model.

Initial *in vitro* studies indicated that the breast carcinoma cell line ZR-75-1 was sensitive to [$AuCl_2$(damp)] (Table 4).[13] This compound was then further tested *in vivo* against a xenograft of the same tumor cells where it demonstrated modest antitumor activity, seen as a reduction in tumor growth compared with controls, comparable to cisplatin. This prompted the synthesis of further analogues with enhanced solubility in order to improve upon the biological properties.[14,16]

The activity of these analogues against the two cell line panels is shown in Tables 4 and 5. The compounds exhibited a cytotoxicity profile similar to cisplatin against the primary cell line panel with the HT1376 bladder carcinoma line being the most sensitive. Interestingly, in the ovarian cell line panel the compounds were equally active against the CH1/CH1-R sensitive/resistant pair.[16]

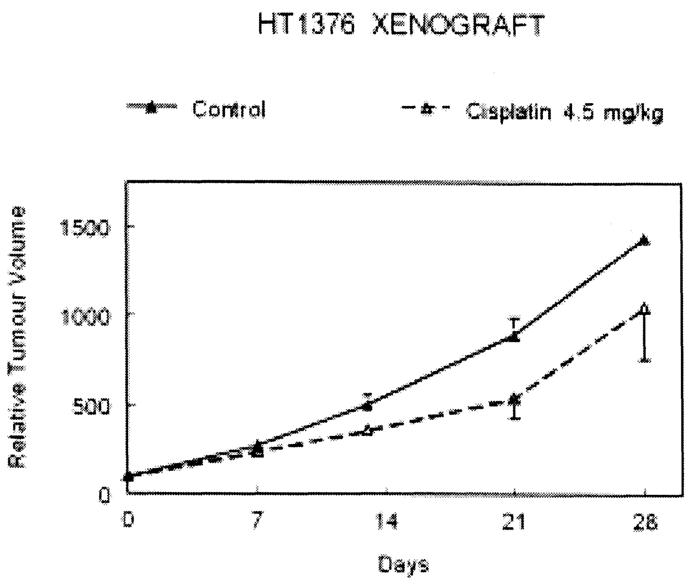

**Figure 2** Growth of the HT1376 bladder carcinoma cell line xenograft. Cisplatin was administered i.p at 0, 7 14, and 21 days. Tumor growth is expressed as Relative Tumor Volume,
(RTV = 100(mean tumor volume at time of assessment)/(mean tumor volume at Day 0)

**Table 4** Cytotoxicity ($IC_{50}$ μM) of Au(III) complexes towards primary tumor cell line panel.

| COMPOUND | SW 620 | SW 1116 | ZR-75-1 | HT29/219 | HT1376 | SK-OV-3 | RANGE |
|---|---|---|---|---|---|---|---|
| **[AuCl$_2$(damp)]** | 124 | 119 | 27 | 55 | 30 | 45 | 4.6 |
| **[Au(SCN)$_2$(damp)]** | 51 | 47 | 34 | 25 | 6.7 | 20 | 7.6 |
| **[Au(OAc)$_2$(damp)]** | 281 | 238 | 45 | 67 | 13 | 13 | 21.6 |
| **[Au(ox)(damp)]** | 205 | 215 | 41 | 19 | 10 | 10 | 21.5 |
| **[Au(mal)(damp)]** | 67 | 80 | 36 | 36 | 11 | 11 | 7.3 |

The complexes (with the exception of [Au(SCN)$_2$(damp)] which had poor solubility and had a similar cytotoxicity profile to the other compounds therefore indicating no activity advantage over the other complexes) were evaluated against the HT1376 tumor *in vivo.*[16] Both the malonato and acetato complexes were active against the HT1376 tumor (see Figures 3 and 4). The chloro complex was less active with the oxalato complex being inactive. This could be explained by their lower solubilities in both water and octanol compared with the acetato and malonato complexes. This poor solubility of the chloro and oxalato complexes would reduce their absorption and limit their biodistribution. The acetato complex [Au(acetato)$_2$(damp)] was also tested against a xenograft of the CH1 ovarian cell line. This compound was also active against this xenograft, though the activity was significantly less than that reported for cisplatin against the same tumor.

**Mechanistic Studies**

Mechanistic studies focused on the acetato complex (Figure 1). Cisplatin was included in the studies in order to compare directly the mode of action of the structurally similar gold(III) complexes. These studies examined the effects of [Au(acetato)$_2$(damp)] at the molecular level by looking at possible interactions with DNA, at the cellular level, and at the pharmacological level in both cell culture and animal models.[16]

**Table 5 Cytotoxicity ($IC_{50}$ μM) of Au(III) complexes towards ovarian tumor cell line panel.**

| COMPOUND | HX62 | SK-OV-3 | CH1 | CH1-R | A2780 | A2780-R | RANGE |
|---|---|---|---|---|---|---|---|
| $[AuCl_2(damp)]$ | 57 | 109 | 13 | 22 (1.7) | 8.2 | 47 (5.7) | 13.3 |
| $[Au(SCN)_2(damp)]$ | 31 | 39 | 10 | 11 (1.1) | 2.0 | 26 (13) | 19.5 |
| $[Au(OAc)_2(damp)]$ | 34 | 107 | 11 | 12 (1.1) | 3.5 | 35 (10) | 31 |
| [Au(ox)(damp)] | 30 | 42 | 11 | 13 (1.2) | 3.7 | 35 (9.5) | 11.4 |
| [Au(mal)(damp)] | 27 | 30 | 2.7 | 3.3 (1.2) | 2.7 | 16 (5.9) | 11.1 |

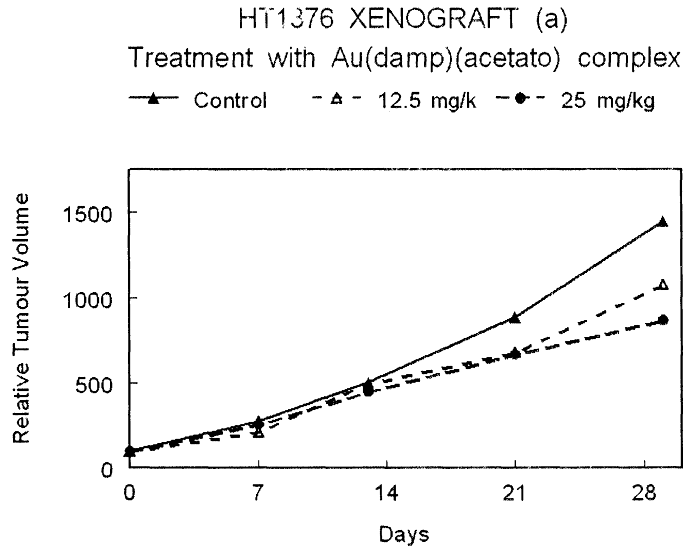

**Figure 3** Growth of the HT1376 bladder carcinoma cell line xenograft after treatment with $[Au(damp)(acetato)_2]$ on Day 0, 7, 14, and 21. Tumor growth is expressed as Relative Tumor Volume (RTV).

The molecular target for cisplatin is DNA.[17,18] Cisplatin can form both inter- and intra-strand cross-links with DNA. The latter, primarily G-G cross-links, are thought to be the cytotoxic lesion. The interaction of $[Au(acetato)_2(damp)]$ with DNA was investigated using two techniques, a plasmid mobility assay, and alkaline elution. The former monitors the migration of a closed circular plasmid, Col E1 in these studies, on an agarose gel.[19] The closed circular plasmid exists in two forms, a supercoiled, condensed form (RFII) and a nicked relaxed form (RFI). The RFII form migrates faster than the RFI form on the gel.

Cisplatin, which can bind to the DNA, reduced the migration of the RFII form. This is due to the induction of inter- and intra-strand cross-links causing unwinding, superwinding, and rewinding of the DNA. The mobility of the RFI form was increased by similar interactions. For $[Au(acetato)_2(damp)]$ no effect was observed on plasmid mobility except at the highest concentration, 1500 μM, 100 fold higher than the $IC_{50}$ against the HT1376 cells. This concentration is so high as to be pharmacologically irrelevant, whereas in contrast cisplatin exerted an effect at <10 μM.

The cross-linking of DNA strands can be assayed using a technique known as alkaline elution.[20] This is a filtration technique in which the formation of DNA cross-links impedes the flow of DNA strands through a filter. This is further enhanced by performing the technique under alkaline conditions, which separates DNA double strands into single strands.

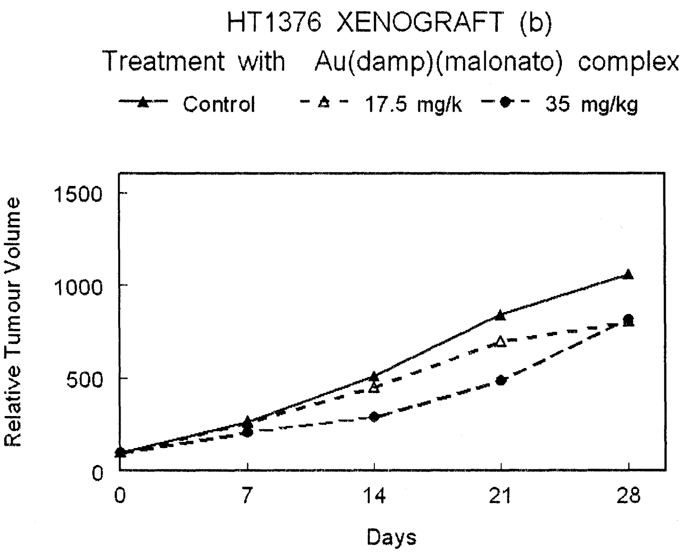

**Figure 4** Growth of the HT1376 bladder carcinoma cell line xenograft after treatment with [Au(damp)(malonato)] on Day 0, 7, 14, and 21. Tumor growth is expressed as Relative Tumor Volume (RTV).

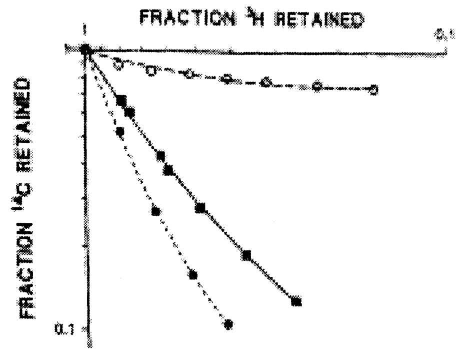

Figure 5a.

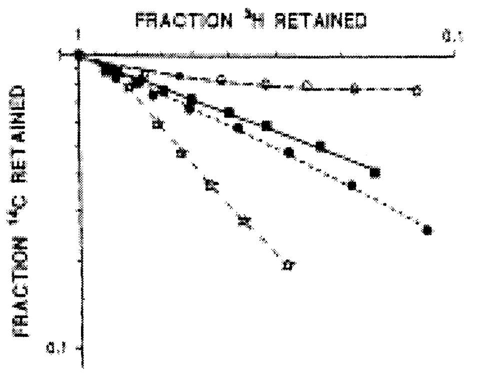

Figure 5b.

| LEGEND |
| --- |
| g Untreated Control |
| o 100μM cisplatin |
| ● 100 μM [Au(damp)(acetate)$_2$] |
| ☆ Irradiated Control |

**Figure 5** Alkaline elution studies in SK-OV-3 cells after exposure to 100 μM [Au(damp)(acetato)$_2$], or 100 μM cisplatin showing formation of (a) interstrand cross-links and (b) DNA strand breaks.

The ovarian tumor cells, SK-OV-3 were incubated for 24 hours in the presence of $^{14}$C-thymidine with either 100 μM cisplatin or 100 μM [Au(acetato)$_2$(damp)]. Cells pre-incubated with $^{3}$H-thymidine were included to provide an internal standard in the assay. These cells were subsequently irradiated to induce DNA strand breaks and included with the treated $^{14}C$-labeled cells. The cells were lysed on the filters and samples collected and analyzed for radioactivity. The results are shown in Figure 5. Cisplatin cross-linked the DNA as shown by the slower rate of elution compared with untreated controls (Figure 5a). Strand-breakage was seen in irradiated, cisplatin treated cells (Figure 5a). The gold complex did not induce DNA cross-links, however there was some indication of DNA strand-breakage (Figure 5a) but this was not evident in the subsequent experiment looking specifically for strand breakage (Figure 5b).

Further evidence that DNA may not be the target for the gold(III) complexes comes from studies on the chemical interactions of these compounds with biological donor ligands. The results indicated that the gold(III) damp complexes had a preference for S-donor ligands such as glutathione and cysteine, with only limited reactivity against nucleosides and their bases.[14]

At the cellular level cisplatin causes a block in the cell cycle at the point where DNA repair occurs, the $S/G_2$ interface. The stages of the cell cycle can be monitored using flow cytometry. In this technique cells are fixed and stained with propidium iodide, a DNA fluorochrome, which binds quantitatively to DNA.[21] The degree of fluorescence is therefore directly proportional to the DNA content of the cell, which in turn is an indicator of the position of the cells in the cell cycle. The ovarian cell line, SK-OV-3 was incubated with $[Au(acetato)_2(damp)]$ for 4 hours at concentrations of either 30 μM or 100 μM and cell cycle analysis determined from DNA content. Using this technique it was apparent that the gold(III) complexes, unlike cisplatin, did not exert a cell cycle specific effect (Table 6).

**Table 6** The effect of $[Au(damp)(acetato)_2]$ on the cell cycle of SK-OV-3 cells. The results show the percentage of cells in the different stages of the cell cycle as determined by flow cytometry.

| | G1 | S | G2 |
|---|---|---|---|
| **CONTROL CELLS** | 43.3 | 41.6 | 15.1 |
| **30 μM $[Au(damp)(acetato)_2]$** | 34.4 | 51.8 | 14.0 |
| **100 μM $[Au(damp)(acetato)_2]$** | 24.4 | 53.8 | 21.9 |

The lack of a cell cycle specific effect was further supported by data from a study looking at the rate of cell kill.[22] The HT1376 bladder carcinoma cells were treated with either cisplatin or $[Au(acetato)_2(damp)]$ for 4 hours over a concentration range of 0-200 μg/ml. Cell survival was then assayed immediately post-dosing (0 hours) and 24, 48 and 72 hours post-dosing. From a comparison of $IC_{50}$ values at different time points (Table 7) it is evident that cisplatin toxicity is delayed, a result of the need for the cells to proceed through a replication cycle in order for cisplatin to exert its effect, compared with $[Au(acetato)_2(damp)]$ where cell death occurs rapidly after dosing.

**Table 7** Rate of cell kill. The HT1376 cells were treated with test compound for 4 hours and cell survival monitored at time intervals post-dosing. Results are expressed as $IC_{50}$ (μg/ml)

| Time post-dosing (hours) | $IC_{50}$ Cisplatin | $IC_{50}$ $[Au(damp)(acetato)_2]$ |
|---|---|---|
| 0 | - | 29 |
| 24 | 48 | 9.5 |
| 48 | 22 | 8.6 |
| 72 | 14 | 8.1 |

The molecular and cellular studies described above indicated that the Au(III)(damp) complexes were acting via a mechanism different to that of cisplatin. These observations were further supported by the *in vitro* and *in vivo* pharmacological data. For example, in the ovarian cell line panel the acetato analogue was equally active against the cisplatin resistant cell line CH1R, as against its cisplatin sensitive counterpart, CH1. The mechanism of resistance to cisplatin in this cell line is repair of cisplatin-induced DNA damage. Also

$[Au(acetato)_2(damp)]$ had little activity against the ADJ/PC6 murine tumor which is known to be particularly sensitive to compounds which cross-link DNA, such as cisplatin.[16]

### Conclusion

It is now apparent that using murine leukemia models as screens for new anticancer drugs has major limitations. This has led to a major reevaluation of tumor screening methodology. The use of human tumor cell lines provides a valuable alternative strategy for identifying new antitumor drugs, including metal-based agents. These cell lines, when incorporated into a panel of selected cell lines, can be used as a primary *in vitro* screen. The ability of many of these cell lines to be grown as xenografts in immune-deprived mice also allows them to be used for *in vivo* evaluation. A mechanistic approach to drug discovery also provides new opportunities for identifying novel agents and should form an integral part of any drug discovery program.

We have adopted an integrated approach combining *in vitro* and *in vivo* tumor models, and investigations into drug mechanism, to evaluate the potential antitumor activity of a class of gold(III) complexes. We have shown that the square planar gold(III) complexes $[Au(acetato)_2(damp)]$ and [Au(malonato)(damp)] have activity against human tumor cells in both *in vitro* and *in vivo* test systems. Detailed studies on their possible mechanism of action at the molecular, cellular and pharmacological level, have shown that these compounds, though superficially similar in structure to cisplatin, act via a quite different mechanism. This observation in particular suggests that these compounds may represent a novel class of metal-based antitumor agent.

### Acknowledgements

This work was the result of a collaboration of a number of scientists. I would like to thank my former colleagues at the Johnson Matthey Technology Centre, Reading, UK; R.G. Buckley, B.R.C. Theobald, A.M. Elsome, and G.R. Henderson; Prof. R.V. Parish and B.P. Howe, UMIST, UK; and L.R. Kelland and his team at the Institute of Cancer Research, Sutton, UK.

### References

1. Corbett, T.H.; Valeriote, F.A.; Baker, L.H. *Invest. New Drugs* **5,** 3-20 (1987).
2. Goldin, A.; Schepartz, S.A.; Venditti, J.M.; Devita, V.T. in *Methods in Cancer Research Vol XVI*; Eds., V.T. Devita Jr., and H. Busch, Academic Press Inc., New York, pp 165-245 (1979).
3. Schwartsmann, G.; Workman, P. *Eur. J. Cancer* **29A**, 3-14 (1993).
4. Boyd, M.R. *Principles and Practice of Oncology Update* **3**, 1-12 (1989).
5. Weinstein, J.N.; Myers, T.G.; O'Connor, P.M.; Friend, S.H.; Fornace, A.J. Jr.; Kohn, K.W.; Fojo, T.; Bates, S.E.; Rubinstein, L.V.; Anderson, N.L.; Buolamwini, J.K.; van Osdol, W.W.; Monks, A.P.; Scudiero, D.A., Sausville, E.A.; Zaharevitz, D.W.; Bunow, B.; Viswanadhan, V.N.; Johnson, G.S.; Wittes, R.E.; Paull, K.D. *Science* **275** 343-349 (1997).
6. Kelland, L.R.; Abel, G.; McKeage, M. J.; Jones, M.; Goddard, P.M.; Valenti, M.; Bryant, A.; Murrer, B.A.; Harrap, K.R. *Cancer Res.* **53**, 2581-2586 (1993).
7. Fricker S.P. in *Metal Ions in Biology and Medicine,* Eds. Ph. Collery, L.A. Poirier, M. Manfait, J-C. Etienne, John Libbey Eurotext, Paris, pp 452-456 (1990).
8. Powis, G. *Curr. Opin. Oncol.* **7**, 554-559 (1995).
9. Scott, G.K. *The Cancer Journal* **10**, 80-86 (1997).
10. Folkmann, J.; *N. Engl. J. Med.* **333**, 1757-1763 (1995).
11. Ni Dhubhghail, O.M.; Sadler, P.J.; in *Metal Complexes in Cancer Chemotherapy*, Ed. B.K. Keppler, VCH, Weinheim, pp 221-248 (1993).
12. Shaw, C.F. III, in *Metal Compounds in Cancer Therapy*, Ed. S.P. Fricker, Chapman and Hall, London, pp 46-64 (1994).
13. Parish, R.V.; Howe, B.P.; Wright, J.P.; Mack, J.; Pritchard, R.G.; Buckley, R.G.; Elsome, A.M.; Fricker, S.P. *Inorg. Chem.* **35**, 1659-1666 (1996).
14. Parish, R.V.; Mack, J.; Hargreaves, L.; Wright, J.P.; Buckley, R.G.; Elsome, A.M.; Fricker, S.P.; Theobald, B.R.C. *J. Chem. Soc. Dalton Trans.* 69-74 (1996).
15. Fricker, S.P.; Buckley, R.G. *Anticancer Res.* **16**, 3755-3760 (1996).
16. Buckley, R.G.; Elsome, A.M.; Fricker, S.P.; Henderson, G.R.; Theobald, B.R.C.; Parish, R.V.; Howe, B.P.; Kelland, L.R. *J. Med. Chem.* **39**, 5208-5214 (1996).
17. Bruhn, S.L.; Toney, J.H.; Lippard, S.J. in *Progress in Inorganic Chemistry: Bioinorganic Chemistry Vol 38*, Ed. S.J. Lippard, John Wiley and Sons, Inc., New York, pp 477-516 (1990).

18. Lempers, E.L.M.; Reedijk, J. *Adv. Inorg. Chem.* **37**, 175-217 (1991).
19. Mirabelli, C.K.; Sung, C-M.; Zimmerman, J.P.; Hill, D.T.; Mong, S.; Crooke, S.T. *Biochem. Pharmacol.* **35**, 1435-1443 (1986).
20. Kohn, K.W.; Ewig, R.A.G.; Erickson, L.C.; Zwelling, L.A. in *DNA Repair - A Laboratory Manual of Research Procedures Vol1 Part B* Eds. E.C. Friedberg, P.C. Hanawalt Marcel Dekker, New York, pp 379-401 (1981).
21. Ormerod, M.G.; Orr, R.M.; Peacock, J.H *Br. J.Cancer* **69**, 93-100 (1994).
22. Elsome, A.M. Ph.D. Thesis, King's College, University of London, UK (1995).

**Received: October 28, 1998 - Accepted in final form: March 7, 1999**

